# Supplementary material for: Peripheral immune landscape for hypercytokinemia in myasthenic crisis utilizing single-cell transcriptomics
Source: J Transl Med. 2023 Aug 24;21:564. doi: 10.1186/s12967-023-04421-y (PMC10464341; doi:10.1186/s12967-023-04421-y)
Supplement: Supplementary file 1 — Additional file 1: Figure S1. Cell distribution before and after batch adjustment. Figure S2. Comparison of cell annotation with prediction results trained from related ScRNA datasets. Figure S3. Cell proportion comparisons between at MC and three months after MC. Figure S4. Overall differentially expressed genes (DEG) analysis at and after MC. Figure S5. Expression comparisons in four monocytes 3 related genes at and after MC. Figure S6. Cell annotation and classification in B and T cells. Figure S7. VDJ combination (immune repertoire) analysis in B and T cells. Figure S8. Cell communications among each cell types (generated in CellChat). Figure S9. Cell communications in each cell types (generated in CellChat). [file 12967_2023_4421_MOESM1_ESM.docx]

**Supplements: Single cell sequencing revealed the peripheral immune landscape for hypercytokinemia in myasthenic crisis**


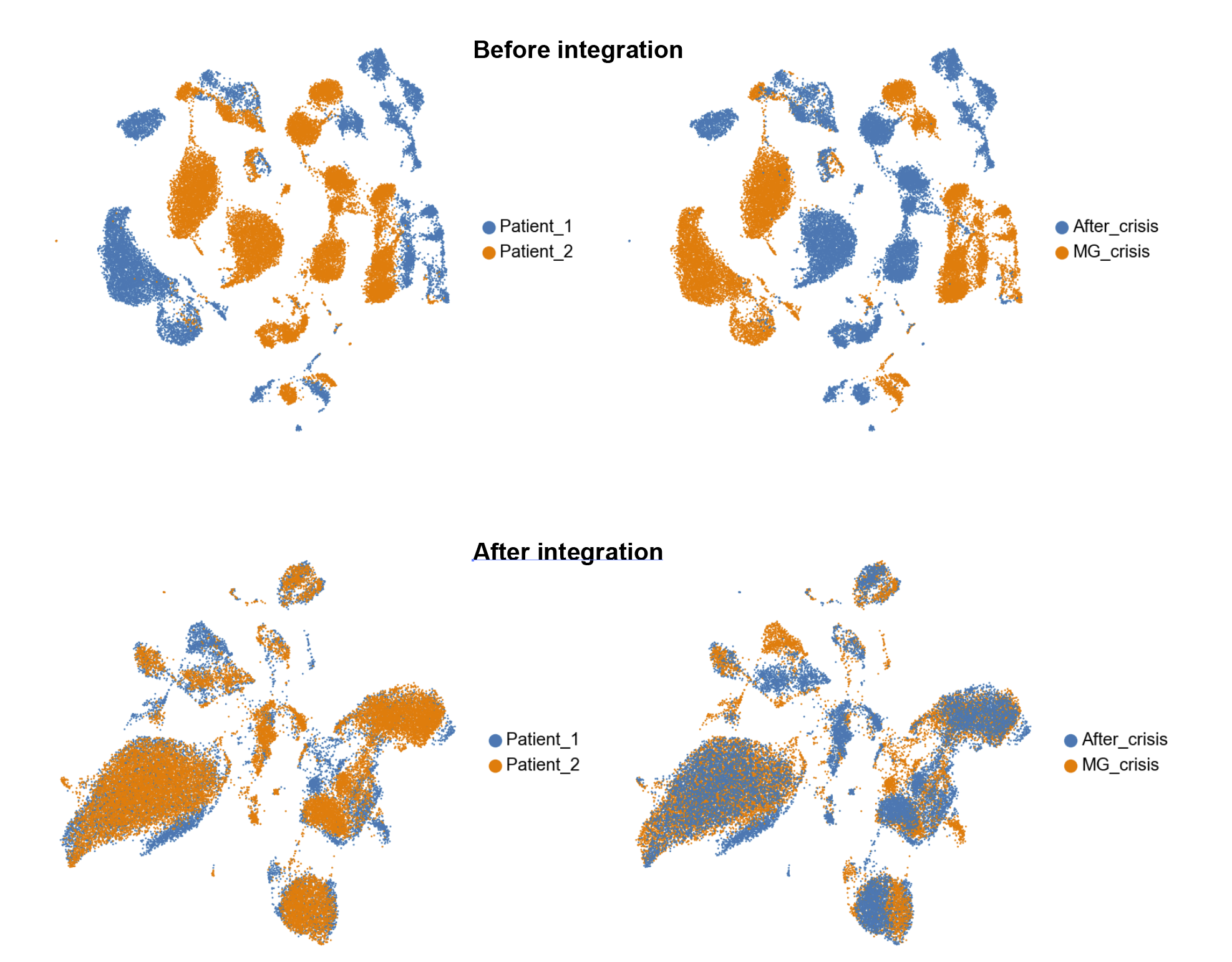


**Figure S1.** **Cell distribution before and after batch adjustment**. The integration of samples from different batches was conducted using “harmony_integrate” function in Scanpy^1^. After batch adjustment, the cells were evenly distributed in each group and phenotype.


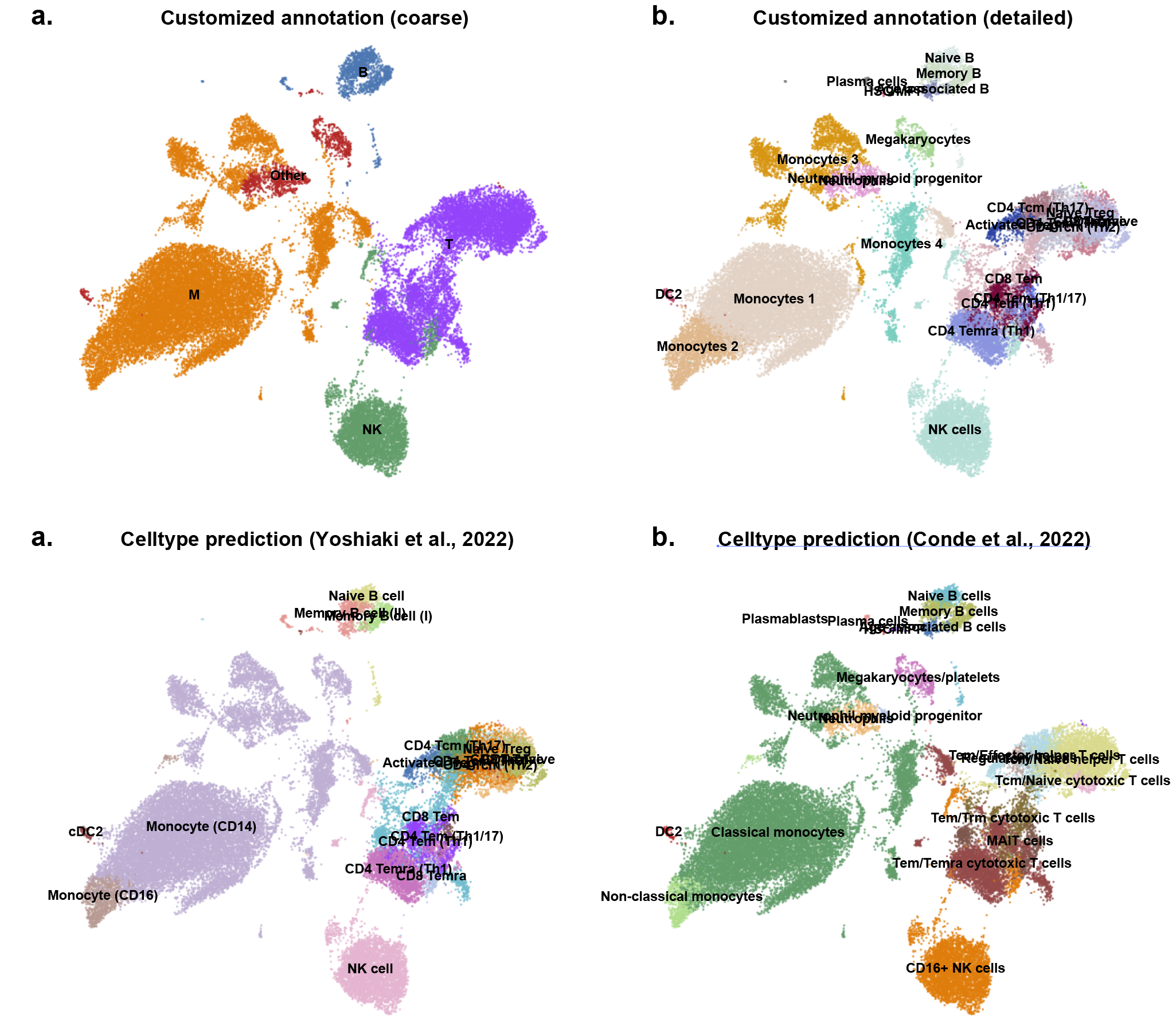


**Figure S2. Comparison of cell annotation with prediction results trained from related ScRNA datasets.** a. Coarse custom cell type annotation. b. Detailed custom cell type annotation. c. Prediction results from a recent Japanese ScRNA dataset related to MG3. The transfer learning utilized a deep-learning-based cell annotation pipeline, Celltypist, to train a new model. The prediction results confirm the classification of the current work. d. Prediction results using a built-in high-resolution immune cell annotation model (Immune_All_Low.pkl) in Celltypist. These prediction results also confirm the classification of the current work.


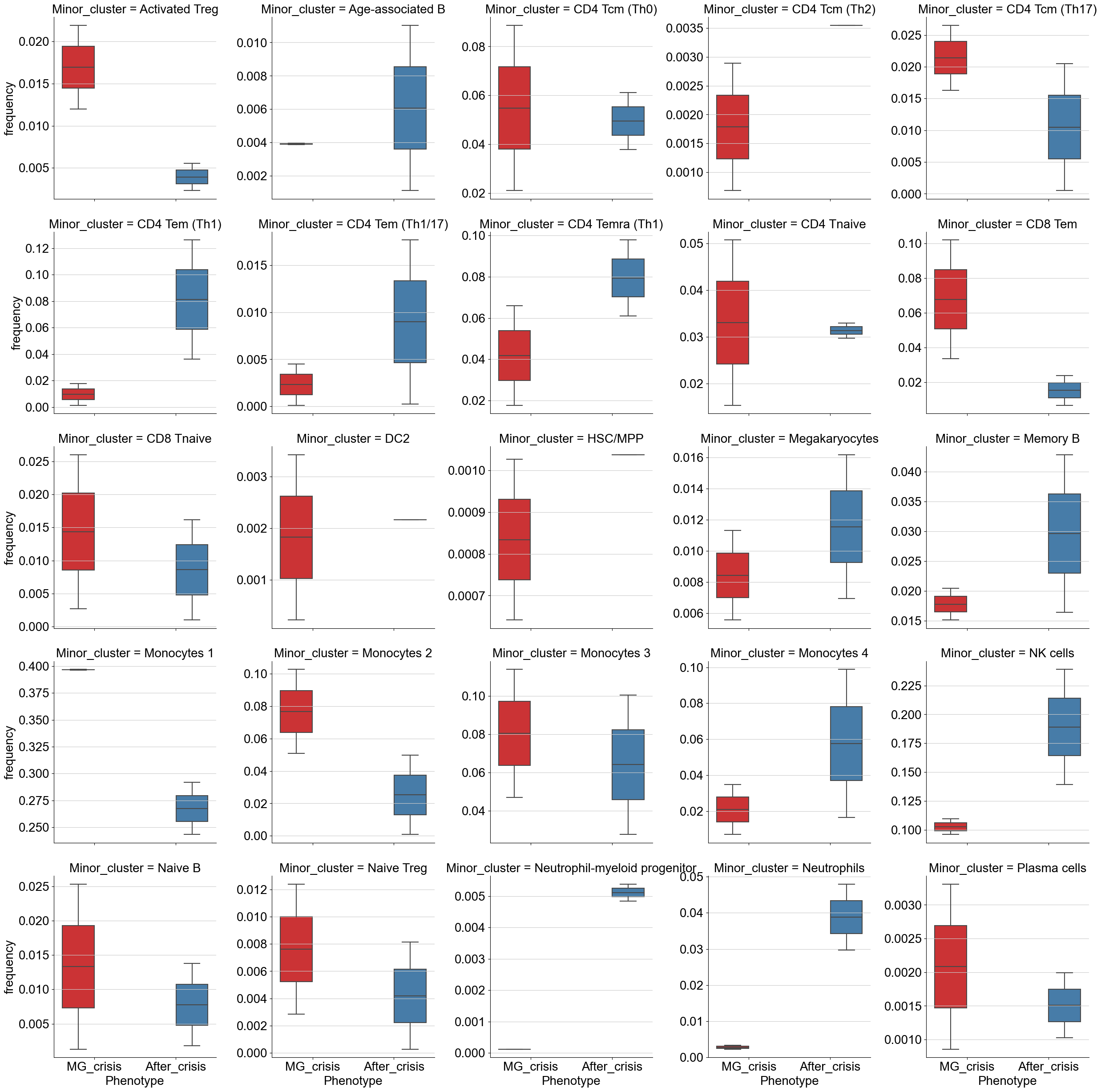


**Figure S3. Cell proportion comparisons between at MC and after MC.** Red is at MC, while blue is three months after MC.


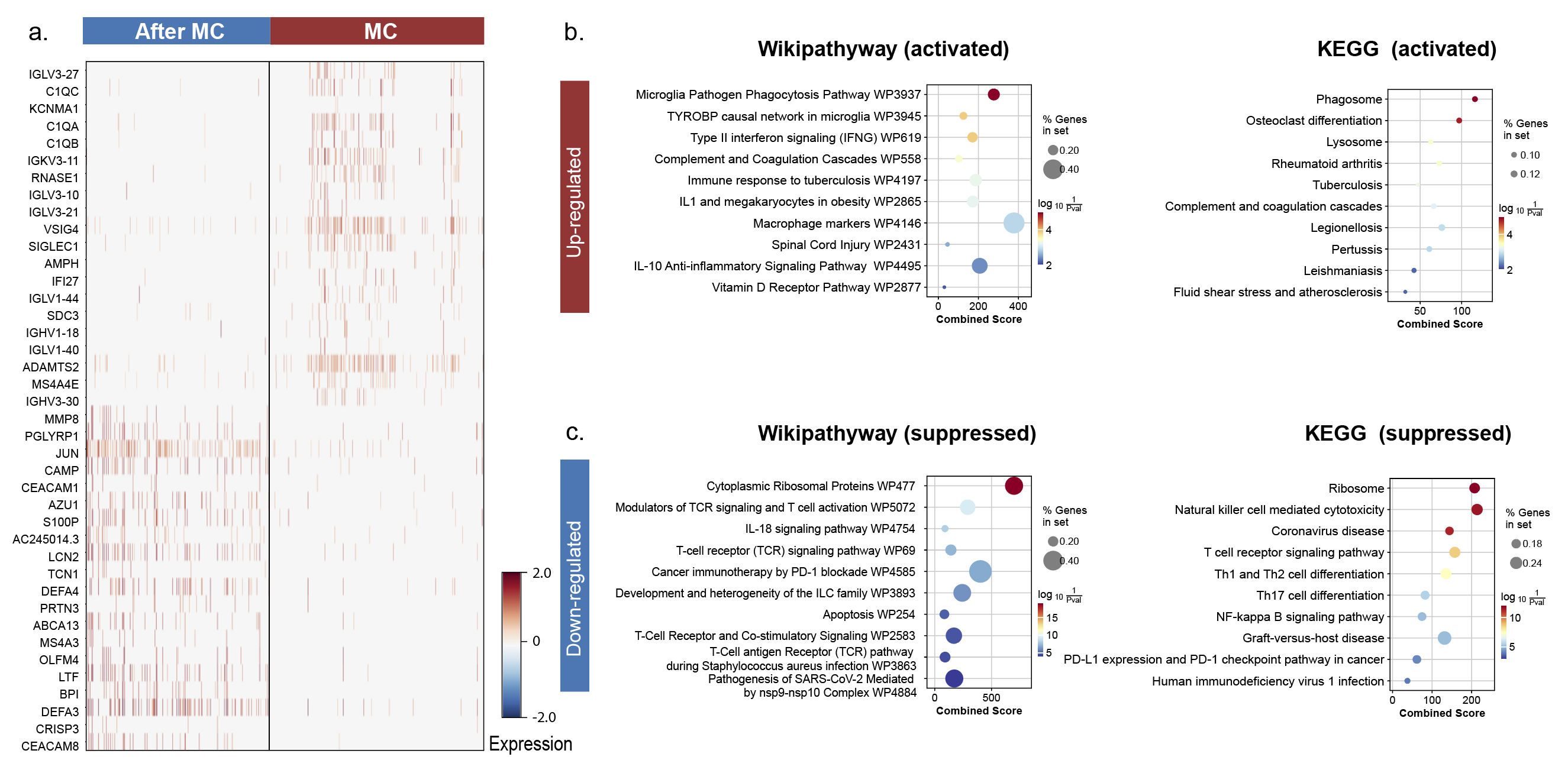
 **Figure S4. Overall differentially expressed genes (DEG) analysis at MC and after MC.** a. The top ranked genes at MC and after MC (three months after discharge). The genes were selected by the absolute values of fold changes. Most MC genes (red) are immune-suppressing genes, indicating the overall immune suppression at MC. b. The enrichment of up-regulated genes at MC enriched in WikiPathway and KEGG database. c. The enrichment of down-regulated genes at MC enriched in WikiPathway and KEGG database.


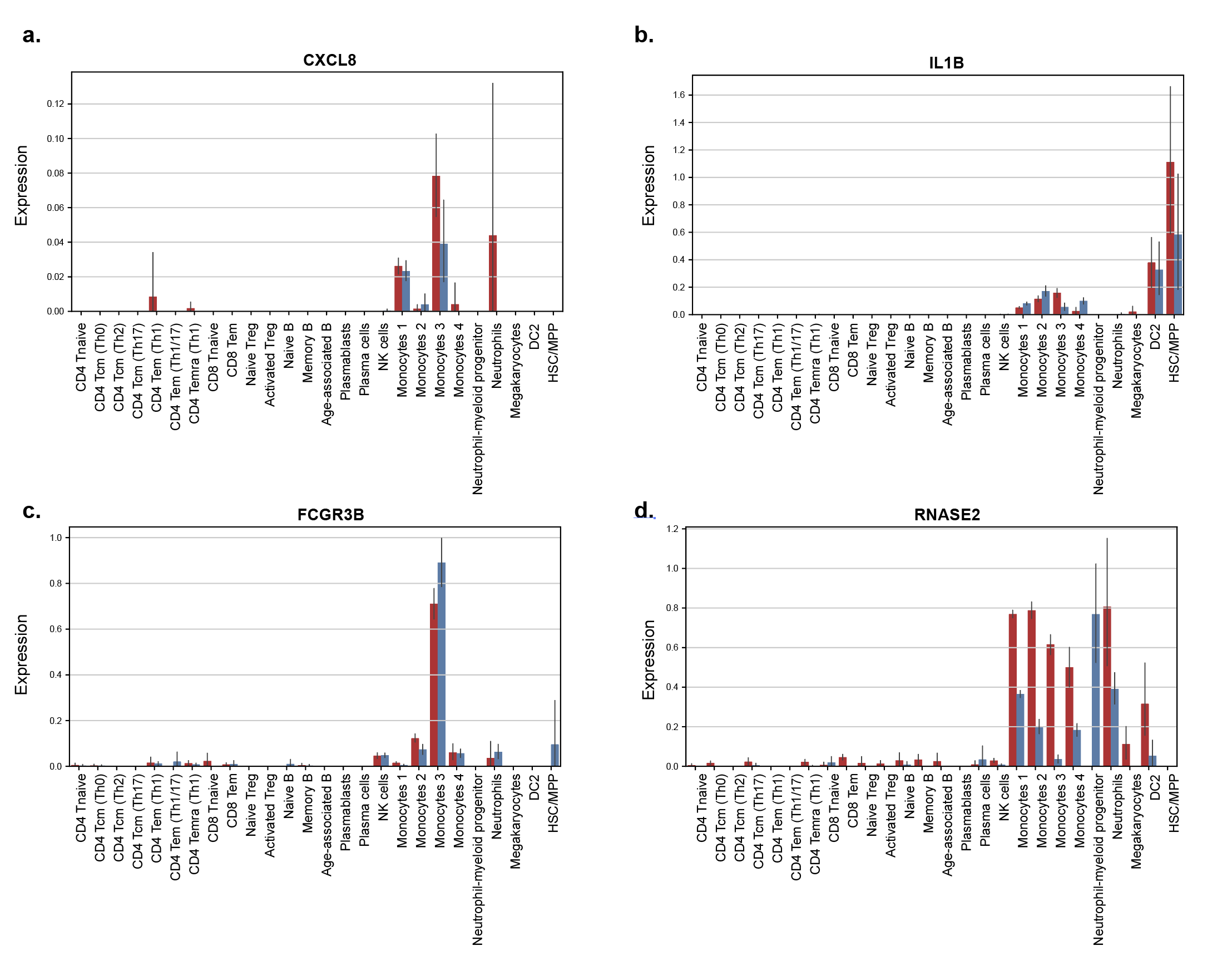


**Figure S5. Expression comparisons in four monocytes 3 related genes at and after MC**. a. Average CXCL8 expressions in monocytes 3. b. Average IL1B expressions in monocytes 3. c. Average FCGR3B expressions in monocytes 3. d. Average RNASE2 expressions in monocytes 3.


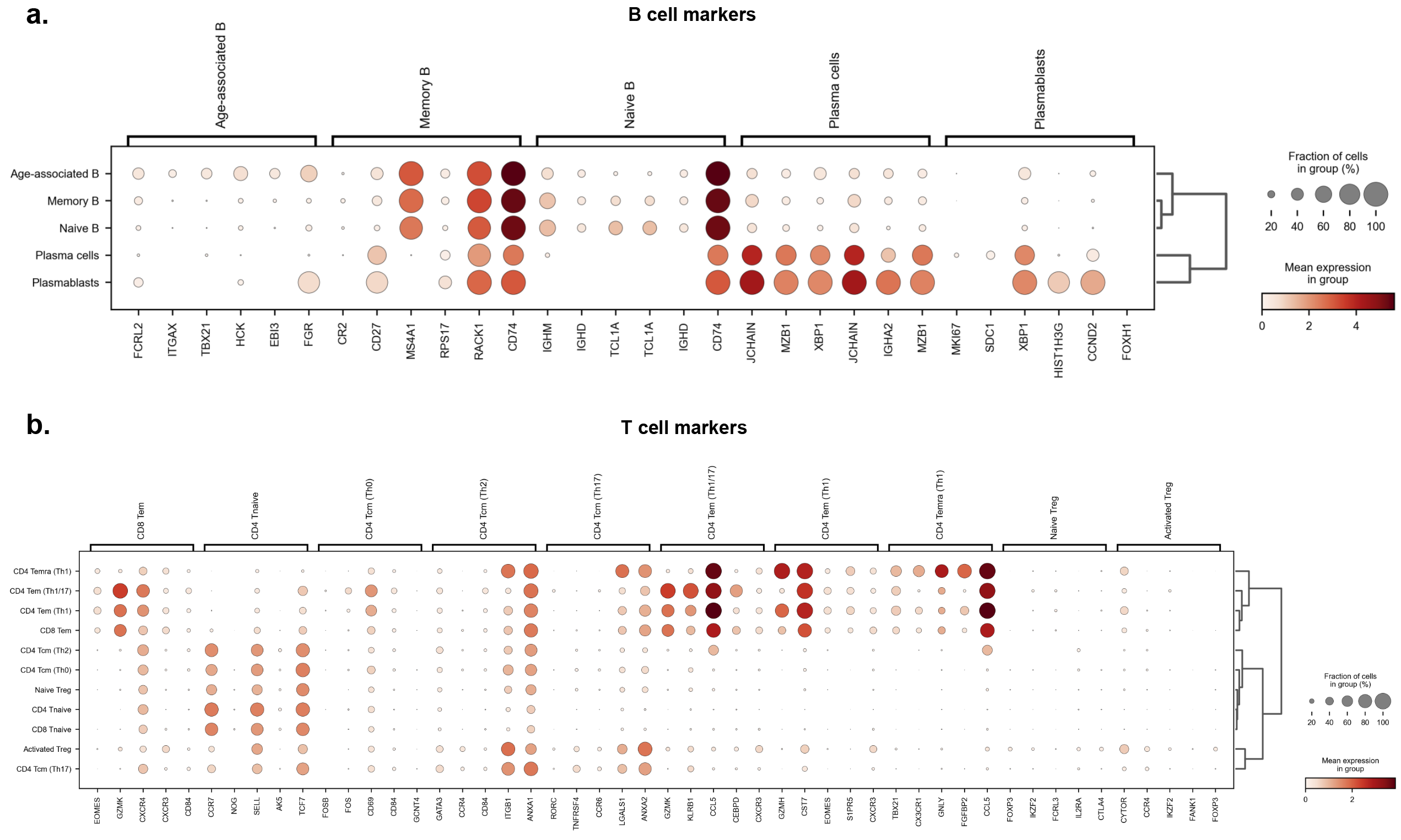


**Figure S6. Cell annotation and classification in B and T cells.** a. The cell markers used to classify B cells. b. The cell markers used to classify T cells.


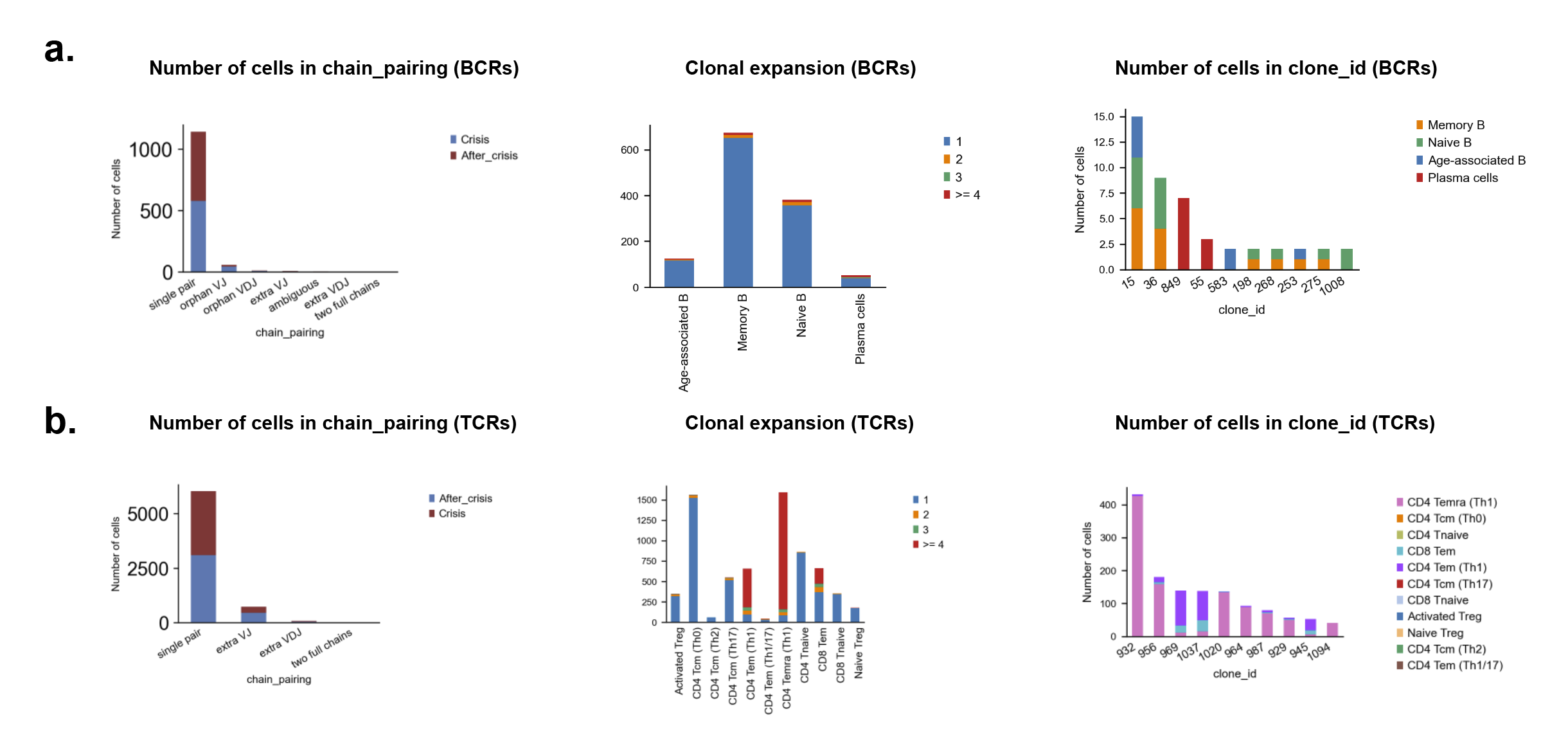


**Figure S7. VDJ combination (immune repertoire) analysis in B and T cells.** a. Only qualified BCRs were used in the analysis. b. Only qualified TCRs were used in the analysis.

BCR, B-cell receptor; TCR, T-cell receptor.


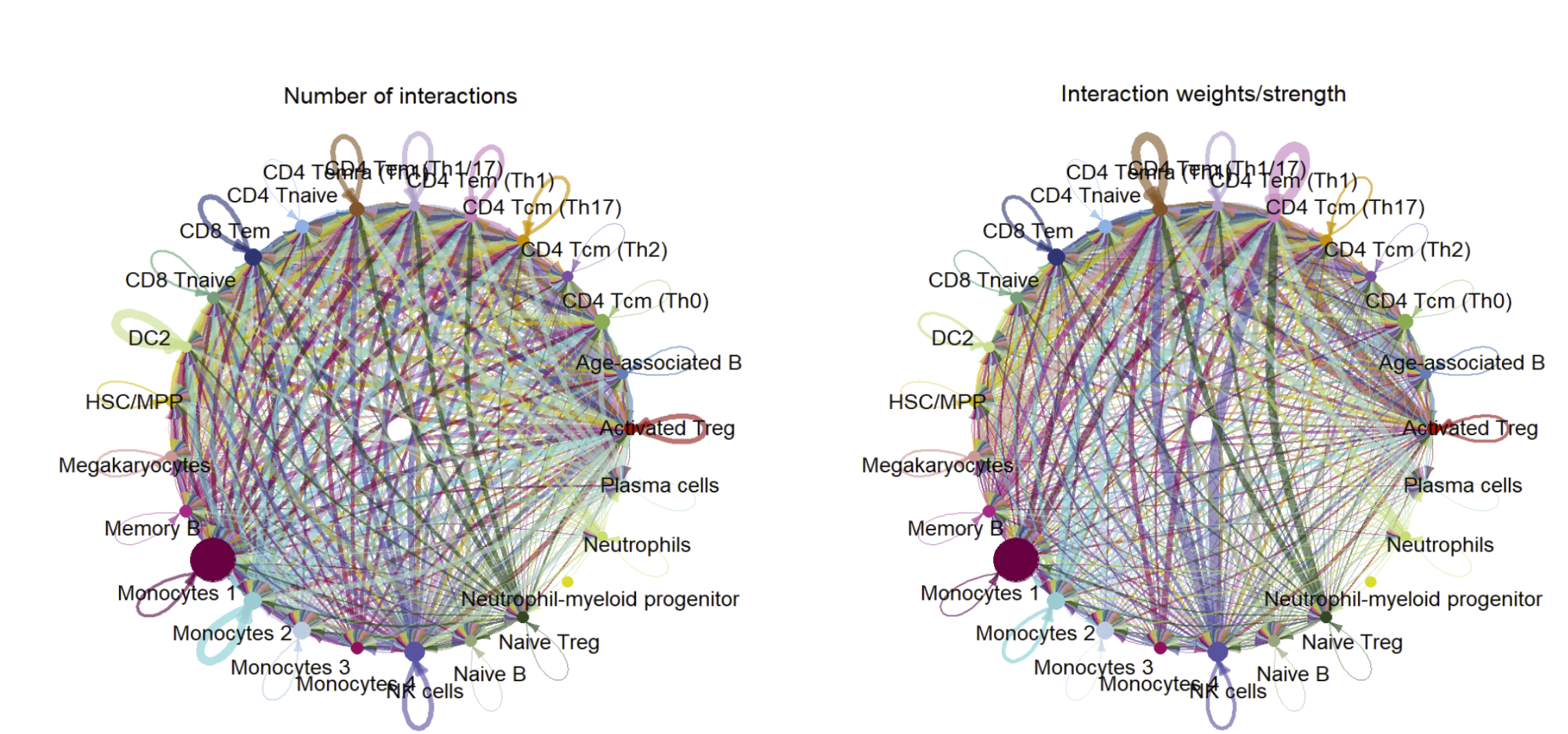
**Figure S8.** Cell communications among each cell types (generated in CellChat^5^). The size of the dot represents the cell proportion, and the thickness of the ribbon represents the strength of the communications.


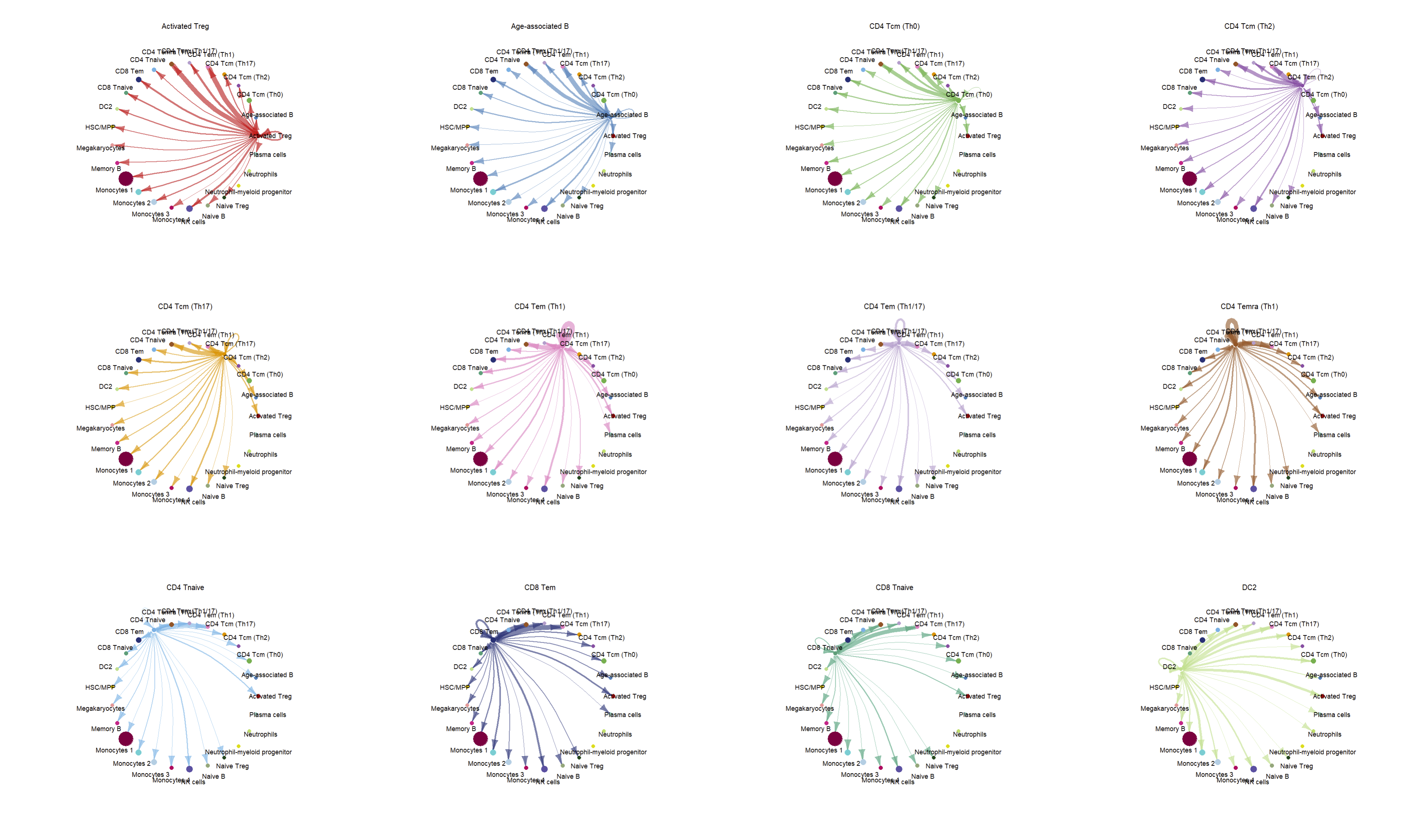

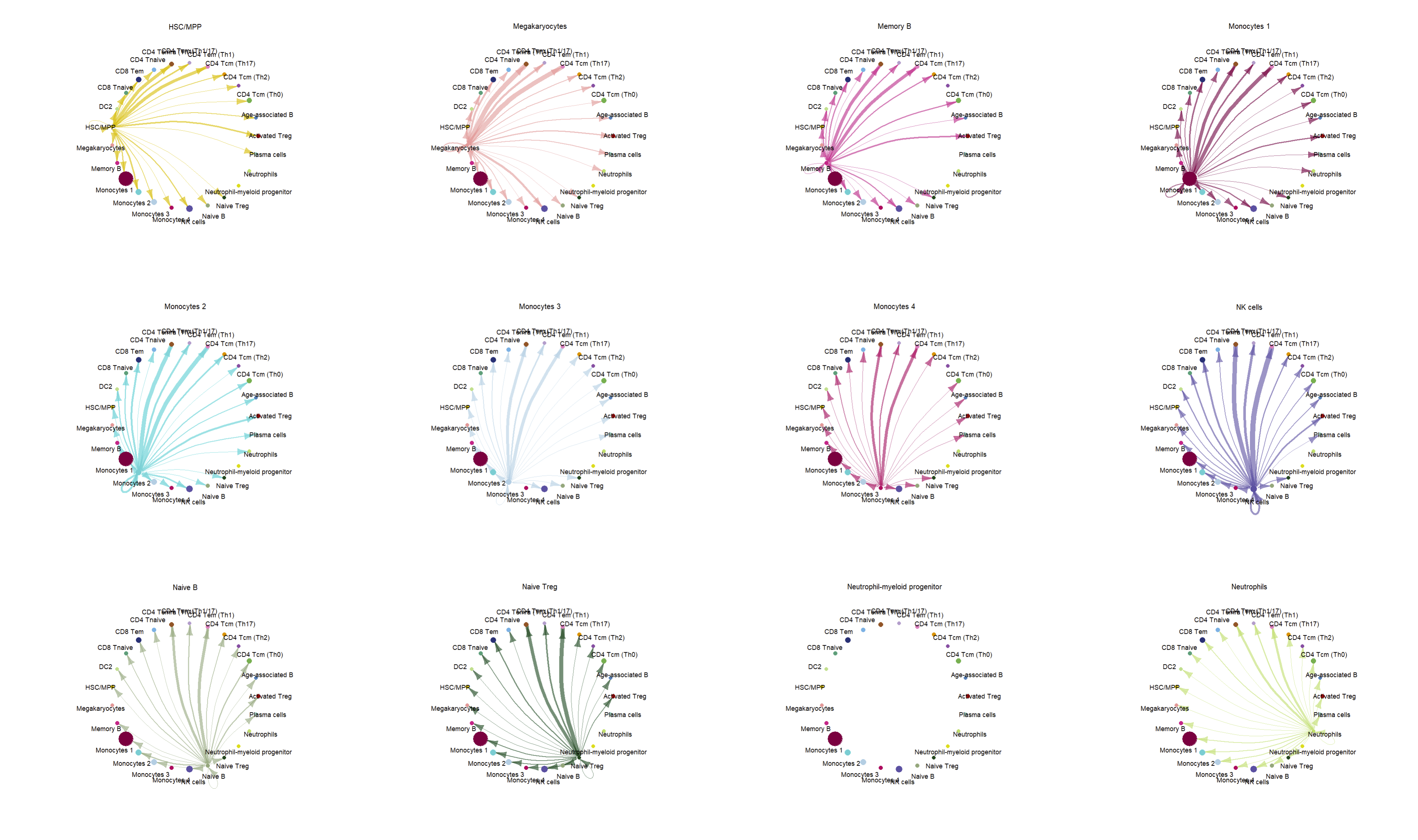

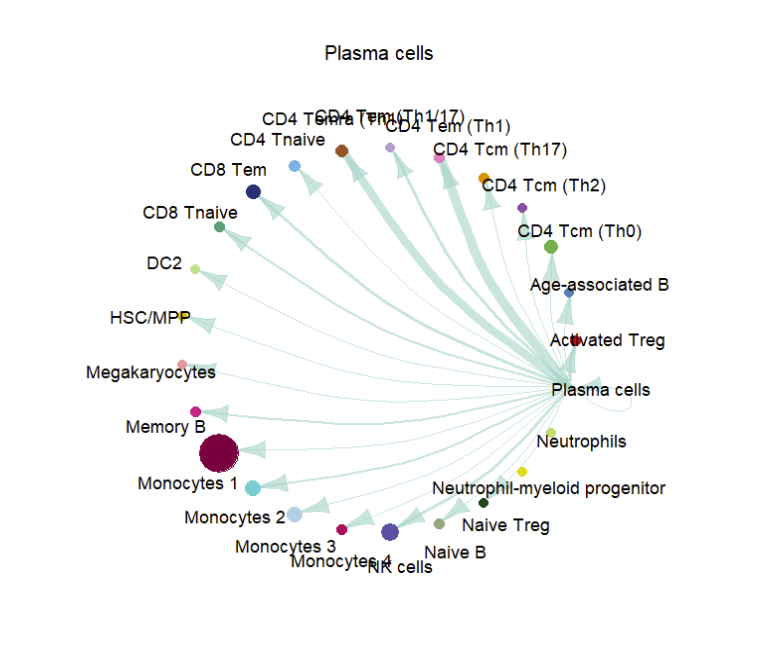


**Figure S9.** Cell communications in each cell types (generated in CellChat).

**References**

1. Korsunsky, I. *et al.* Fast, sensitive and accurate integration of single-cell data with Harmony. *Nat Methods* **16**, 1289–1296 (2019).

2. La Manno, G. *et al.* RNA velocity of single cells. *Nature* **560**, 494–498 (2018).

3. Yasumizu, Y. *et al.* Myasthenia gravis-specific aberrant neuromuscular gene expression by medullary thymic epithelial cells in thymoma. *Nat Commun* **13**, 4230 (2022).

4. Domínguez Conde, C. *et al.* Cross-tissue immune cell analysis reveals tissue-specific features in humans. *Science* **376**, eabl5197 (2022).

5. Jin, S. *et al.* Inference and analysis of cell-cell communication using CellChat. *Nat Commun* **12**, 1088 (2021).
